# Th17 expansion in granulomatosis with polyangiitis (Wegener's): the role of disease activity, immune regulation and therapy

**DOI:** 10.1186/ar4066

**Published:** 2012-10-18

**Authors:** Benjamin Wilde, Marielle Thewissen, Jan Damoiseaux, Marc Hilhorst, Pieter van Paassen, Oliver Witzke, Jan Willem Cohen Tervaert

**Affiliations:** 1 Department of Internal Medicine, Division of Clinical and Experimental Immunology, University Hospital Maastricht, PO Box 5800, Maastricht, 6202 AZ, The Netherlands; 2 Department of Nephrology, University Duisburg-Essen, Hufelandstr. 55, Essen, 45127, Germany; 3 Laboratory of Clinical Immunology, Maastricht University Medical Center, PO Box 5800, Maastricht, 6202 AZ, The Netherlands

## Abstract

**Introduction:**

In autoimmune diseases, IL-17 producing T-cells (Th17), a pro-inflammatory subset of T-cells, are pathophysiologically involved. There is little knowledge on the role of Th17 cells in granulomatosis with polyangiitis (GPA). In the present study, we investigated Th17 cells, Tregs and subsets of circulating Th17 cells in GPA and related results to disease activity.

**Methods:**

42 GPA patients in remission, 18 with active disease and 14 healthy controls (HC) were enrolled. Th17 cells, their subsets and regulatory T-cells were determined by intracellular fluorescence activated cell sorter (FACS). Data are given as mean percentage ±SD of total T-helper-cells.

**Results:**

Th17 cells are expanded in active and quiescent GPA as compared to HC (1.7±1.4% vs. 0.7 ±0.3%, *P *= 0.006 and 1.9 ±1.5% vs. 0.7 ±0.3%, *P*<0.0001). Th17 expansion is stable over time and does not decline when remission is achieved. However, a negative association of Th17 cells and steroid dosage is observed (r=-0.46, *P *= 0.002). The Th17 expansion was not balanced by Tregs as indicated by skewed Th17/Treg ratios in active and quiescent GPA. Th17 subsets co-producing IFNγ or IL-10 are significantly increased in GPA. GPA patients in remission not receiving maintenance therapy have significantly more IL-10/IL-17A double positive T-cells than HC (0.0501 ±0.031% vs. 0.0282 ±0.016%, *P *= 0.007).

**Conclusions:**

We provide evidence for a persistent, unbalanced expansion of Th17 cells and Th17 subsets which seems to be independent of disease activity. Maintenance therapy reduces -but does not normalize- Th17 expansion.

## Introduction

Granulomatosis with polyangiitis (GPA) is an autoimmune form of necrotizing small-vessel vasculitis characterized by the presence of antineutrophil cytoplasmic antibodies (ANCA). GPA is strongly associated with ANCA directed against proteinase-3 (PR3) and in a minority of cases, ANCA with specificity for myeloperoxidase (MPO) are detected [1-3]. Alongside the role of the autoantibodies in disease pathogenesis, T cells also contribute to disease mechanisms as suggested by the isotype of ANCA, which indicates that a T cell-dependent class-switch has taken place [4]. Furthermore, T cells are present in inflammatory lesions related to GPA and it is thought that granuloma formation in GPA is T cell-dependent [5-7]. Accordingly, deviation of circulating T cells has been described in GPA showing persistent T cell activation, expansion of memory T cells and a deficient function of regulatory T cells [8-14].

Th17 cells were recently described as a separate T-helper-cell lineage with distinct features [15,16]. IL-17A is a signature cytokine of this T-helper-cell lineage. IL-17A drives inflammation by promoting neutrophil/macrophage recruitment, enhances auto-antibody production and facilitates tissue destruction by upregulation of matrix metalloproteases [17,18]. A role for Th17 cells has been established in autoimmune diseases such as giant cell arteritis (GCA), rheumatoid arthritis (RA) and systemic lupus erythematosus (SLE) [17,19-22]. In these diseases, increased circulating Th17 cells have been closely associated to disease activity.

Also in GPA, a pathogenic role of Th17 cells is likely. Gan *et al*. showed in an animal model of experimental MPO-vasculitis that IL-17A deficiency attenuated the disease [23]. In addition, the frequency of auto-antigen-specific Th17 cells in convalescent ANCA-vasculitis is increased [24,25]. Another study by Fagin *et al*. showed that auto-antigen-specific Th17 cells, including IL-17A/IFNγ and IL-17A/IL-4 double producers, were also increased in active ANCA-vasculitis [26]. The role of auto-antigen-specific T cells in the initiation of autoimmunity is controversial. Ghani *et al*. found that antigen-specific T cells condition inflamed sites for high-rate antigen non-specific effector T cell recruitment [27]. In contrast, Murakami *et al*. recently reported that antigen specificity is of limited importance to initiate autoimmune processes [28]. In an experimental mouse model, activated Th17 cells were able to initiate arthritis in the absence of cognate antigen recognition [28]. Since comparable non-antigen-dependent mechanisms might be operative in GPA, we investigated 1) the presence of circulating Th17 cells in active and quiescent GPA, 2) whether Th17 expansion is balanced by regulatory T-cell subsets, and 3) concomitant expression of different cytokines in Th17 cells.

## Materials and methods

### Patient cohort

We enrolled 60 patients with GPA, (12 with MPO-ANCA, 48 with PR3-ANCA, Table 1), mean age (± SD) 57 (± 13) years, and 14 age-matched healthy volunteers (healthy controls, HC), mean age 53 ± 5 years. Eighteen patients were in an active state of disease at presentation and forty-two were in remission (Table 1, 2). Informed patient consent and approval by the local ethics committee was obtained. Seven patients initially presenting with active disease were assessed again after remission was achieved. The diagnosis and classification of GPA was made according to criteria of the American College of Rheumatology, the Chapel Hill criteria and the algorithm published by Watts *et al*. [29-31]. Active disease was defined as clinical manifestation of new-onset or recurrent disease activity related to vasculitis requiring intensified immunosuppressive therapy [32,33]. All patients with active disease were either untreated or treated with maintenance therapy at the time of sampling. None of the active patients had received cyclophosphamide or high dose intravenous methylprednisolone at the time of sampling. Remission or quiescent disease was defined as absence of clinical disease activity reflecting a Birmingham Vasculitis Activity Score of zero [33]. The cohort of patients in remission was divided for further analysis into two groups: patients in long-term stable remission, and patients with a relapsing disease course. Patients with a relapse-free disease course since the first onset of GPA and a minimum disease duration of 48 months, were defined as being in stable long-term remission (n = 13). Patients with a minimum disease duration of 48 months and at least one relapse within the first 48 months since diagnosis of GPA were defined as relapsers (n = 19).

**Table 1:** Clinical characteristics of the patient cohort

Characteristic	Remission	Active disease
Number	42	18
Age, years, mean ± SD	58 ± 13	54 ± 14
PR3/MPO	37/5	11/7
Disease duration, months, mean ± SD	105 ± 75	67 ± 97
Treatment, yes/no	16/26	7/11
Steroids, yes/no	13/3 (12 patients: 5 to 10 mg; 1 patient: 30 mg)	7/0 (4 patients: 5 to 10 mg; to 45 mg)
AZA/MMF/MTX/CYC	7/2/4/0	2/1/0/0

PR3/MPO, proteinase-3/myeloperoxidase (describes the anti-neutrophil-cytoplasmic antibodies specificity at the time of diagnosis); AZA, azathioprine; CYC, cyclophosphamide; MMF, mycophenolate mofetil; MTX, methotrexate.

**Table 2:** Characteristics of patients with active disease at the time of sampling

Patient number	Age (years)	ANCA type	Disease duration (months)	Disease activity at time of sampling	BVAS	CRP (mg/L)
1	55	PR3	0	A, ENT, K, L	25	292
2	37	PR3	44	A, ENT	5	20
3	55	PR3	57	A, K, L	15	247
4	58	MPO	120	A, L	7	23
5	49	PR3	190	A, ENT, L	10	46
6	57	PR3	259	Ey, ENT	6	< 5
7	56	PR3	191	Ey	6	< 5
8	82	MPO	0	L	4	44
9	53	PR3	0	A, ENT, K, L	17	160
10	74	PR3	0	K	10	NA
11	69	PR3	9	ENT, K	14	211
12	41	MPO	0	A, ENT, K, S	17	44
13	58	MPO	0	ENT, K, S	14	116
14	70	MPO	0	A, ENT, K, L	19	188
15	53	PR3	15	Ey, K	12	12
16	24	PR3	9	A, Ey	3	16
17	40	MPO	33	ENT	6	< 5
18	41	MPO	284	K, L	12	< 5

None of the patients received induction therapy at the time of sampling. PR3/MPO, proteinase-3/myeloperoxidase; this reflects anti-neutrophil-cytoplasmic antibodies specificity (ANCA) at the time of diagnosis. Organ involvement of patients with active disease at the time of sampling is depicted. A, arthritis (joints); BVAS, Birmingham Vasculitis Activity Score; CRP, C-reactive protein; ENT, ear-nose-throat; Ey, ophthalmologic manifestation of disease; K, kidney; L, lung; S, skin.

### Flow cytometry: staining of surface antigens

Lymphocyte phenotypes were measured by five-colour surface staining. The following antibodies labeled with fluorescent dye were used: CD3 (mouse IgG1, HorV450), CD4 (mouse IgG1, PerCP; ITK/Biolegend, Uithoorn, The Netherlands), CD8 (mouse IgG1, APC-H7), CD25 (mouse IgG1, FITC), CD127 (mouse IgG1, PE). All antibodies except CD4 were purchased at BD Bioscience, Breda, The Netherlands. Appropriate isotype controls (BD Bioscience) were used. Peripheral blood mononuclear cells (PBMC) were isolated by standard Ficoll density gradient centrifugation (Histopaque; Sigma Aldrich, Zwijndrecht, The Netherlands) and incubated with labeled monoclonal antibodies for 30 minutes in the dark at room temperature. Analysis was performed with a fluorescence-activated cell sorter (FACS) CANTO™ from BD Bioscience.

### Immunostaining for intracellular cytokines

PBMC from patients and HC were separated by standard Ficoll density gradient centrifugation. The cells were resuspended in RPMI 1640 medium (Gibco Invitrogen, Breda, The Netherlands) supplemented with 10% heat-inactivated fetal calf serum (Greiner Bio-One, Alphen a/d Rijn, The Netherlands), 100 U/mL penicillin and 100 µg/mL Streptomycin (both Gibco Invitrogen). The cells were cultured in the absence or presence of PMA (50ng/mL) and Ionomycin (1µg/mL) (Sigma-Aldrich) for 4 hours. Cytokine secretion was inhibited by Brefeldin A (BD Bioscience). Surface staining was performed with CD3 HorV450 and CD8 APC-H7 (both BD Bioscience). Cells were fixed using a Cytofix/Cytoperm kit (BD Bioscience). Finally, the samples were stained intracellularly for CD69 (ITK/Biolegend), IFNγ (BD Bioscience), IL-4, IL-10 and IL-17A (all ITK/Biolegend). Unstimulated PBMC and appropriate isotype controls were used to confirm specificity of staining and to discriminate background staining. FSC-H vs. FSC-A gating allowed exclusion of cell doublets. As stimulation with PMA/ionomycin induced downregulation of CD4, CD4**^+ ^**T cells were defined as CD3**^+^**CD8**^neg^**, and an average of 30,000 events was acquired in this CD3**^+^**CD8**^neg ^**gate. CD69 expression was used as a control for the activation procedure but was not included in the gating strategy. CD69 expression did not differ between HC and patients with GPA after mitogen stimulation (94.97 ± 3.49% vs. 95.25 ± 3.54%, *P *> 0.05).

### Statistics

All values are expressed as mean ± SD. Significance for the differences between groups was determined using the Mann-Whitney *U*-test. Spearman's rank correlation test was applied to detect correlation between different study parameters.

## Results

### Circulating Th17 cells are increased during active disease and remain elevated during remission

**Figure 1:**
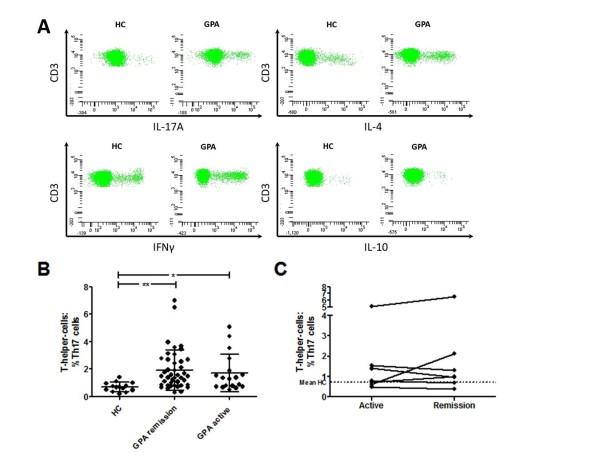
**Th17 cells in cross-sectional and longitudinal analysis**. (**A**) Representative fluorescence activated cell sorter (FACS) plots of a volunteer healthy control (HC) and a patient with granulomatosis with polyangiitis (GPA). Plots are gated on CD3**^+^**CD8**^neg ^**T cells and 40,000 events are shown in each graph. (**B**) The percentage of Th17 cells was higher in quiescent GPA than in HC. Active GPA patients showed a significantly increased Th17 cell fraction compared to HC and did not differ from quiescent GPA. Mean values are indicated by the horizontal line, and error bars represent the SD. (**C**) Seven patients were first assessed during the active phase of disease and then again after remission had been achieved. The dotted line represents the mean value in the HC. *P < 0.05, **P < 0.005.

Patients with active GPA (n = 18) showed a significantly increased percentage of circulating IL-17A**^+ ^**T cells compared to HC (n = 14) (% of CD4**^+ ^**T cells = 1.7 ± 1.4% vs. 0.7 ±0.3%, *P *= 0.006 (Figure 1A, B), while there was no difference in IFNγ (14.5 ± 9.9% vs. 17.7 ± 7.9%, *P *= 0.17), or IL-4-producing CD4**^+ ^**T cells (5.6 ± 3.3% vs. 4.9 ± 2.1%, *P *= 0.7). There were no significant differences between active patients with or without maintenance therapy at the time of sampling.

Th17 expansion was similar between active GPA and quiescent GPA (n = 42, 1.7 ± 1.4% vs. 1.9 ± 1.5%, *P *= 0.37 (Figure 1B). Seven patients first assessed during the active state of disease were sampled again during remission (median time between visits 118 days, range 71 to 567 days). The percentage of Th17 cells did not decline during remission (Figure 1C).

Accordingly, circulating IL-17A**^+ ^**T cells were elevated in GPA patients in remission compared to HC (% of CD4**^+ ^**T cells = 1.9 ± 1.5% vs. 0.7 ± 0.3%, *P *< 0.0001) (Figure 1A, B) whereas IFNγ (22.1 ± 10.8% vs. 17.7 ± 7.9%, *P *= 0.20), and IL-4 producing-T-helper-cells (6.1 ± 2.5% vs. 4.9 ± 2.1%, *P *= 0.11) did not differ between patients with GPA and the HC. Of note, IFNγ^+ ^T-cells were significantly reduced in patients with active GPA compared to those with quiescent disease (14.5 ± 9.9% vs. 22.1 ± 10.8%, *P *= 0.002).

CD3**^+^**CD4**^neg^**CD8**^neg ^**(DN) T-cells were previously described as producers of IL-17 [34]. However, there was no difference between HC and patients with GPA in the percentage of DN T cells (% of CD3**^+ ^**T-cells in the HC vs. quiescent GPA = 2.9 ± 2.0% vs. 3.1 ± 2.9%, *P *> 0.05, and in the HC vs. active GPA = 2.9 ± 2.0% vs. 3.4 ± 4.1%, *P *> 0.05). Moreover, DN T cells did not correlate with Th17 cells (*r *= -0.4, *P *= 0.2 in the HC; *r *= -0.3, *P *= 0.3 in patients with GPA).

### Clinical associations with Th17 cells in GPA

ANCA-status, that is, PR3/MPO-ANCA negative (n = 12) vs. positive (n = 30) at the time of sampling, did not have impact on the percentage of Th17 cells (data not shown). Patients with (n = 16) or without maintenance (n = 26) therapy both had a significant increase of Th17 cells compared to the HC (1.33 ± 0.94% vs. 0.7 ± 0.3%, *P *= 0.03 and 2.32 ± 1.61% vs. 0.7 ± 0.3%, *P *< 0.0001). However, patients on maintenance therapy harbored a significantly smaller fraction of Th17 cells than untreated patients (1.33 ± 0.94% vs. 2.32 ± 1.61%, *P *= 0.01).

In addition, administration of steroids had an impact on Th17 cells as there was a negative correlation of steroid dosage and percentage of Th17 cells (*r *= -0.46, *P *= 0.002). A similar effect was observed for Th1 cells and steroid dosage (*r *= -0.32, *P *= 0.04), and so a separate analysis accounting for maintenance therapy was also performed. To assess the impact of Th17 cells on the clinical course of the disease, we stratified the patient cohort into relapse-free and relapsing patients, and identified 13 relapse-free patients and 19 relapsing patients. Two relapse-free patients and nine relapsing patients received maintenance treatment at the time of sampling. As shown before, treatment seemed to have impact on the percentage of Th17 cells. Therefore, we compared untreated relapsing patients in remission (n = 10) with untreated relapse-free patients in remission (n = 11). Th17 cells were similar between both groups (% Th17 cells = 2.3 ± 1.0% vs. 2.6 ± 2.2%, *P *= 0.7).

### Disturbed balance of circulating Th17 cells and regulatory T cells in active and quiescent GPA

**Figure 2:**
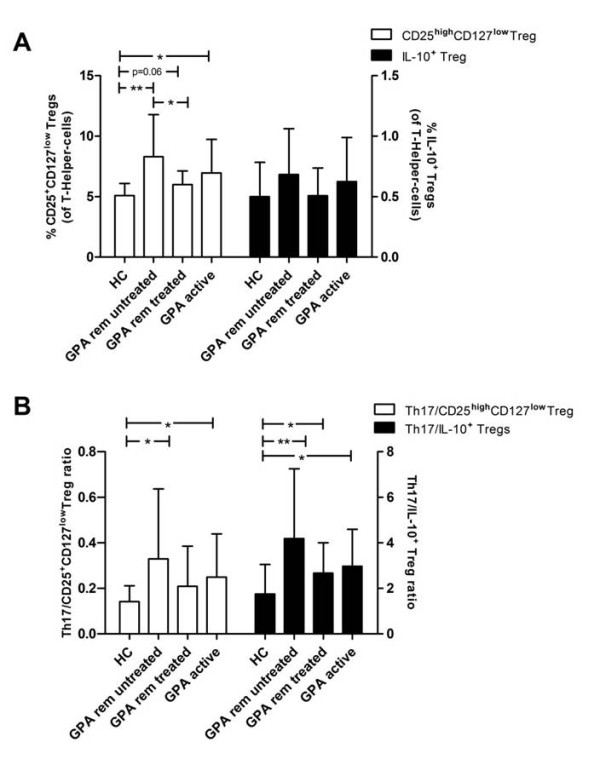
**Regulatory T cells (Tregs) are expanded in granulomatosis with polyangiitis (GPA) but do not balance Th17 skewing**. (**A**) The percentage of IL-10**^+ ^**Tregs was similar in active/quiescent GPA vs. healthy controls (HC), whereas CD25**^high^**CD127**^low ^**Tregs were significantly increased. (**B**) The Th17/IL-10**^+ ^**Treg ratio was significantly increased in quiescent untreated GPA (4.18 ± 3.07, *P *= 0.001, n = 26), quiescent treated GPA (2.67 ± 1.34, *P *= 0.02, n = 16) and active GPA (2.97 ± 1.63, *P *= 0.04, n = 17) vs. HC (1.75 ± 1.30, n = 14). The Th17/CD25**^high^**CD127**^low ^**Treg ratio was higher in quiescent, untreated GPA (0.33 ± 0.31, *P *= 0.008, n = 22) and in active GPA (0.25 ± 0.19, *P *= 0.04, n = 18), but not in quiescent, treated GPA (0.21 ± 0.18, *P *= 0.54, n = 13) compared to HC (0.14 ± 0.07, n = 13). Bars represent means, error bars indicate the SD. *P < 0.05, **P < 0.006.

Regulatory T-helper-cell subsets control and limit immune responses of pro-inflammatory T cells. In this study, we focused on two types of regulatory T cells (Tregs), which were defined according to literature: CD4**^+^**CD25**^high^**CD127**^low ^**Tregs and IL-10**^+ ^**Tregs [35,36]. CD4**^+^**CD25**^high^**CD127**^low ^**Tregs were increased in quiescent GPA (8.3 ± 3.5% in untreated patients, n = 22; 6.0 ± 1.1% in treated patients, n = 13) and active GPA (7.0 ± 2.8%, n = 18) compared to the HC (5.1 ± 1.0%, n = 13)(*P *< 0.006, *P *= 0.06, and *P *= 0.02 respectively, for comparison with the HC) (Figure 2A). In contrast, IL-10**^+ ^**Tregs did not differ between quiescent GPA (0.7 ± 0.4% in untreated patients, n = 26; 0.5 ± 0.2% in treated patients, n = 16) or active GPA (0.6 ± 0.4%, n = 17) compared to the HC (0.5 ± 0.3%, n = 14) (*P *= 0.13, *P *= 0.7, and *P *= 0.52 respectively, for comparison with the HC) (Figure 2A). As Th17 cells and Treg share developmental pathways, a reciprocal relationship has been described [37]. Accordingly, Th17 expansion is associated with Treg depletion in human SLE [22]. Therefore, a correlation analysis was performed to assess the Th17/Treg relationship in GPA. Th17 cells were positively correlated with CD4**^+^**CD25**^high^**CD127**^low ^**or IL-10**^+ ^**Tregs in quiescent GPA (*r *= 0.34, *P *< 0.05 and *r *= 0.38, *P *= 0.01) and active GPA (*r *= 0.36, *P *= 0.14 and *r *= 0.60, *P *= 0.01) but not in the HC (*r *= -0.04, *P *= 0.90 and *r *= 0.19, *P *= 0.52). Separate analysis of GPA patients in remission stratified by maintenance treatment showed only a significant positive correlation between IL-10**^+ ^**Tregs and Th17 in patients on maintenance therapy (*r *= 0.68, *P *= 0.004). Thus, a reciprocal relationship or a negative association between Th17 cells and Tregs was not found.

Ratios were calculated to assess if the balance between Th17 cells and Tregs was preserved in GPA. Importantly, Th17 expansion was not balanced by Tregs in quiescent, untreated GPA and active GPA indicated by both a higher Th17/CD25**^high^**CD127**^low ^**Treg ratio and Th17/IL-10**^+ ^**Treg ratio compared to HC (Figure 2B). Interestingly, in quiescent, treated GPA, only the Th17/IL-10**^+ ^**Treg ratio was significantly skewed towards Th17, whereas the Th17/ CD25**^high^**CD127**^low ^**Treg ratio was not different from HC (Figure 2B). Relapsing and relapse-free patients had comparable Th17/Treg ratios, with 0.36 ± 0.32 vs. 0.39 ± 0.38 (*P *= 1.0) for Th17/CD25**^high^**CD127**^low ^**Treg, and 4.9 ± 2.8 vs. 4.3 ± 3.8 (*P *= 0.6) for Th17/IL-10**^+ ^**Treg.

### Th17 cells co-expressing IL-10 or IFNγ are elevated in active GPA and untreated quiescent disease

**Figure 3:**
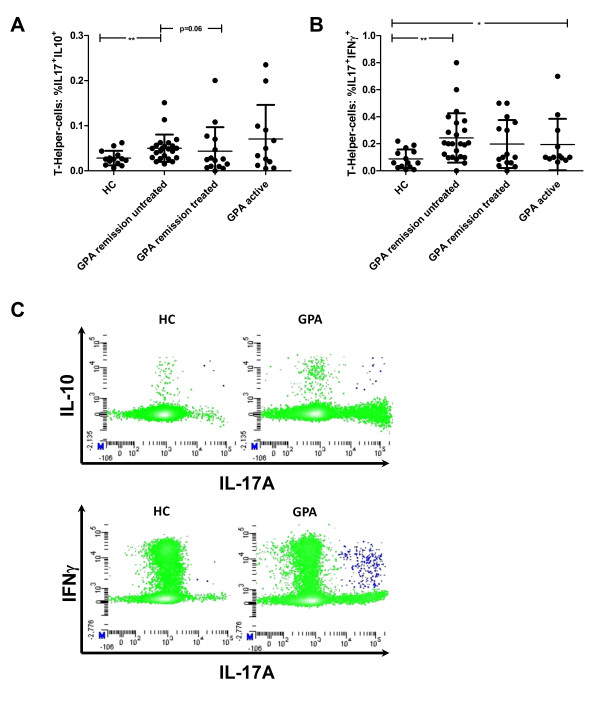
**Th17 cell subsets are increased in granulomatosis with polyangiitis (GPA)**. (**A**) IL-17**^+^**IL-10**^+ ^**CD4**^+ ^**T cells were increased in quiescent, untreated GPA compared to quiescent, treated GPA and healthy controls (HC). No difference was detected between HC and patients with active GPA. (**B**) IFNγ producing Th17 cells were increased in quiescent, untreated GPA and active GPA compared to HC. Means are indicated by the horizontal line, and the error bars represent the SD. (**C**) Representative fluorescence-activated cell sorter (FACS) plots of a HC and a GPA patient. IL-10 and IFNγ producing Th17 cells are depicted in the upper right corner (blue). Plots are gated on CD3**^+^**CD8**^neg ^**T cells, 30,000 events were acquired for each sample in this gate. *P < 0.05, **P < 0.008.

IFNγ and IL-10 co-expression of Th17 cells was measured in a subset of GPA patients. Both IL-10**^+^**/IL-17A**^+ ^**and IFNγ**^+^**/IL-17A**^+ ^**CD4**^+ ^**T cells could be detected in GPA and HC. The percentage of IL-10**^+^**/IL-17A**^+ ^**CD4**^+ ^**T cells was higher in untreated quiescent GPA (0.0501 ± 0.031%, n = 23), but not in treated quiescent GPA (0.0432 ± 0.053%, n = 15), than in the HC (0.0282 ± 0.016%, n = 14) (*P *= 0.007 and *P *= 0.91 respectively, for comparison with the HC) (Figure 3A).

In active GPA, the percentage of the IL-10**^+^**/IL-17A**^+^**population (0.0703 ± 0.076%, n = 12) did not differ from the HC (0.0282 ± 0.016%, n = 14, *P *> 0.05) (Figure 3A).

IFNγ**^+^/**IL-17A**^+^**CD4**^+ ^**T cells were significantly increased in untreated, quiescent GPA (0.24 ± 0.18%, n = 24, *P *= 0.001), but not in treated, quiescent GPA (0.20 ± 0.18%, n = 15, *P *= 0.09), and in active GPA (0.19 ± 0.19%, n = 12, *P *< 0.05) compared to the HC (0.09 ± 0.07%, n = 14) (Figures 3B and 3C). IFNγ**^+^**/IL-10**^+ ^**T cells did not differ between the patients and HC (data not shown). Thus, Th17 cells producing IL-10 or IFNγ were increased only in untreated, quiescent and active GPA.

## Discussion

In patients with GPA, we observed Th17 expansion, and demonstrated that Th17 expansion is present during active disease and persists after remission has been achieved. Th17 cells seemed to be sensitive to maintenance treatment but remained elevated. Th17 expansion was not balanced either by CD25**^high^**CD127**^low^**, or by IL-10**^+ ^**Tregs. Finally, we observed an elevated percentage of Th17 cells expressing IL-10 or IFNγ in untreated GPA, which possibly indicates enhanced plasticity.

Antigen-specific Th17 expansion has previously been reported in GPA [24,26,38]. The role of antigen-specific T-cells in initiation of tissue inflammation is controversial. According to Ghani *et al*., antigen-specific T-cells are crucial initiators of tissue inflammation, whereas a recent publication by Murakami *et al*. suggests that antigen specificity of Th17 cells is of limited importance for the initiation of autoimmune processes [27,28]. Therefore, both auto-antigen-specific and, as determined in our study, non-specific Th17 expansion may be relevant in GPA. The driving force of Th17 cell expansion remains unclear; chronic challenge with auto-antigen or deficient function of Tregs seems likely [24,25,39]. Alternatively, high levels of cytokines promoting Th17 polarization could be an underlying cause. In line with this, increased levels of the Th17 enhancing cytokines TGFβ, IL-6 and IL-23 can be found in the sera of GPA [24,40]. DN T-cells have been recently described as one major source of IL-17 and seem to be expanded in SLE [34]. However, in GPA patients, levels of DN T-cells appeared similar to those in the HC, and Th17 cells did not correlate with DN T-cells. Therefore, the nature of Th17 expansion might be different in SLE when compared to GPA.

We found similar, elevated levels of circulating Th17 cells both in patients with active and quiescent GPA, implying that there was no association with disease activity. This is in contrast to findings in SLE and giant cell arteritis (GCA) where a close relationship of IL-17A**^+ ^**T cell numbers and disease activity is reported [19,22,41]. Interestingly, IFNγ**^+ ^**T-cells were significantly diminished during active GPA compared to quiescent GPA, suggesting an inverse association with disease activity. IFNγ**^+ ^**T-cells are found in inflamed lesions in GPA and recruitment from the circulation may therefore explain the reduction during the active phase of the disease [10,42-44]. In GCA, steroid therapy during active disease reduces Th17 cell numbers very efficiently and the reduction is maintained during remission [19]. Indeed, in our study a negative association between steroid dose and Th17 cells was observed, indicating some sensitivity to steroids. However, in contrast to GCA, a persistent reduction to normal levels is not achieved in GPA. Th17 cells remained elevated in quiescent GPA patients on maintenance therapy, and even more so in GPA patients in whom maintenance therapy was previously withdrawn. This might have implications for the disease course. There is circumstantial evidence that persistent Th17 expansion is associated with a higher tendency to relapse. In a study by Nogueira *et al*., five patients were prospectively followed, of whom two showed elevated IL-17A serum levels after remission was achieved. Only those two patients relapsed within 15 months after first presentation, whereas the other three remained in remission (follow-up time 15, 20 and 23 months) [24]. In our study, however, we could not link Th17 expansion to relapse propensity.

The skewing towards Th17 was not sufficiently balanced by CD4**^+^**CD25**^high^**CD127**^low ^**or IL-10-producing Tregs. A disturbed balance of pro-inflammatory and anti-inflammatory T cell subsets with a shift towards pro-inflammatory subsets is suggested to be an important pathogenic mechanism of autoimmunity [45]. Accordingly, the observed shift of the Th17/Treg ratio in GPA might point to an increased risk for exaggerated immune responses in case of trigger events such as infections [46]. Indeed, infections may provoke relapses in patients with GPA [47,48]. To a certain extent, patients on maintenance therapy showed a normalization of the Th17/Treg ratio.

We detected an increased fraction of IL-17**^+^**IFNγ**^+ ^**and IL17**^+^**IL-10**^+ ^**T cells in GPA. IL-17**^+^**IFNγ**^+ ^**are described in human studies as a very potent pro-inflammatory T cell subset with enhanced migratory capacity [49-51]. Thus, IL-17**^+^**IFNγ**^+ ^**might have a role in tissue inflammation in GPA. Moreover, it is known that T cells have the capacity to convert into other T cell lineages [50,52]. Plasticity of Th17 and Treg might be an important factor in autoimmune diseases [53]. Protective Treg converting into harmful Th17 cells could promote pro-inflammatory conditions and be fatal for immune homeostasis [54]. Plasticity is a transient, dynamic process and double-positive IL-17**^+^**IL-10**^+ ^**T cells can be found temporarily during conversion as part of this process [53]. The exact role and significance of these Th17 subsets remains to be studied. [21,49,50,53].

In conclusion, we provide evidence for a persistent, unbalanced expansion of Th17 cells and Th17 cell subsets which is partly reversed by maintenance therapy in GPA. Our findings may indicate a pathogenic role of Th17 cells in GPA.

## Conclusions

Th17 cells and Th17 subsets are expanded in GPA irrespective of disease activity. Furthermore, the balance of Th17 cells and Tregs seems to be disturbed. Maintenance therapy does not normalize Th17 expansion, which persists after therapy is ceased. The significance of Th17 subsets producing IFNγ and/or IL-10 should be studied further.

## Abbreviations

ANCA: anti-neutrophil-cytoplasmic antibodies; FACS: fluorescence-activated cell sorter; GCA: giant cell arteritis; GPA; granulomatosis with polyangiitis; HC: healthy controls; IFNγ: interferon gamma; MPO: myeloperoxidase; PBMC: peripheral blood mononuclear cells; PMA: phorbol-12-myristat-13-acetate; PR3: proteinase-3; RA: rheumatoid arthritis; SLE: systemic lupus erythematosus; TGFβ: transforming growth factor beta; Th17 cells: T-helper-17-cells; Treg: regulatory T cell.

## Competing Interests

The authors declare that they have no competing interests.

## Authors' contributions

All authors contributed to the design, acquisition and interpretation of data. BW performed the statistical analysis. BW, MT, MH, JD, PvP, OW and JWCT drafted the manuscript. BW carried out flow cytometry. BW and JWCT assessed and participated in the interpretation of the clinical data. All authors read and approved the final manuscript.
